# Neurotransmitters Modulate Intrathymic T-cell Development

**DOI:** 10.3389/fcell.2021.668067

**Published:** 2021-04-13

**Authors:** Carolina Francelin, Luciana Peixoto Veneziani, Alessandro dos Santos Farias, Daniella Arêas Mendes-da-Cruz, Wilson Savino

**Affiliations:** ^1^Autoimmune Research Laboratory, Department of Genetics, Microbiology and Immunology, Institute of Biology, University of Campinas, Campinas, Brazil; ^2^National Institute of Science and Technology on Neuroimmunomodulation (INCT-NIM), Oswaldo Cruz Institute, Oswaldo Cruz Foundation, Rio de Janeiro, Brazil; ^3^Department of Ophthalmology and Visual Sciences, School of Medicine, University of Alabama at Birmingham, Birmingham, AL, United States; ^4^Laboratory on Thymus Research, Oswaldo Cruz Foundation, Oswaldo Cruz Institute, Rio de Janeiro, Brazil; ^5^Rio de Janeiro Research Network on Neuroinflammation (RENEURIN), Rio de Janeiro, Brazil; ^6^School of Pharmacy and Biomedical Sciences, University of Central Lancashire, Preston, United Kingdom

**Keywords:** neurotransmitters, thymus, thymic epithelial cells, T-cell development, thymocytes

## Abstract

The existence of a crosstalk between the nervous and immune systems is well established. Neurotransmitters can be produced by immune cells, whereas cytokines can be secreted by cells of nervous tissues. Additionally, cells of both systems express the corresponding receptors. Herein, we discuss the thymus as a paradigm for studies on the neuroimmune network. The thymus is a primary lymphoid organ responsible for the maturation of T lymphocytes. Intrathymic T-cell development is mostly controlled by the thymic microenvironment, formed by thymic epithelial cells (TEC), dendritic cells, macrophages, and fibroblasts. Developing thymocytes and microenvironmental cells can be influenced by exogenous and endogenous stimuli; neurotransmitters are among the endogenous molecules. Norepinephrine is secreted at nerve endings in the thymus, but are also produced by thymic cells, being involved in controlling thymocyte death. Thymocytes and TEC express acetylcholine receptors, but the cognate neurotransmitter seems to be produced and released by lymphoid and microenvironmental cells, not by nerve endings. Evidence indicates that, among others, TECs also produce serotonin and dopamine, as well as somatostatin, substance P, vasoactive intestinal peptide (VIP) and the typical pituitary neurohormones, oxytocin and arg-vasopressin. Although functional data of these molecules in the thymus are scarce, they are likely involved in intrathymic T cell development, as exemplified by somatostatin, which inhibits thymocyte proliferation, differentiation, migration and cytokine production. Overall, intrathymic neuroimmune interactions include various neurotransmitters, most of them of non-neuronal origin, and that should be placed as further physiological players in the general process of T-cell development.

## Introduction

The concept of neuroimmune crosstalk was established several decades ago. One key factor in determining such communication is the fact that both the nervous and immune systems use similar molecular moieties, and therefore apply a common syntax to communicate with each other. Accordingly, classic types of neurotransmitters can be produced by immune cells, whereas cytokines can be secreted by cells of nervous tissues. In the same vein, cells from the two systems express the correlated receptors, although signal transduction may be specific for a given cell type. Herein, we discuss the thymus as a paradigm for the expression and role of different kinds of neurotransmitters, particularly of non-neuronal origin.

**FIGURE 1:**
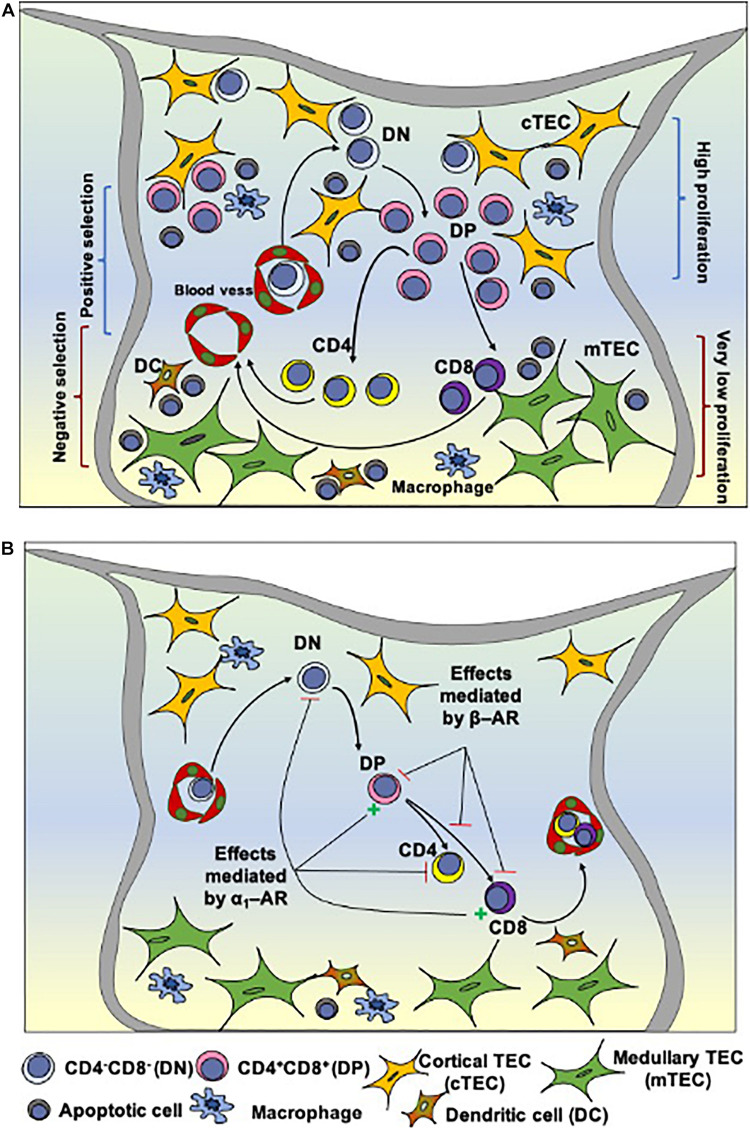
Intrathymic T cell differentiation within the thymic microenvironment and stages that can be modulated by norepinephrine. **(A)** Shows schematically the general process of thymocyte differentiation, in the context of the thymic microenvironment. Bone marrow-derived precursors enter the organ through capillaries at the corticomedullary junction and migrate toward the outer cortex where they proliferate, but do not express the CD3/TCR complexes as well as the accessory molecules CD4 and CD8. There CD4/CD8 double-negative cells (DN) evolve to express TCR as well as CD4 and CD8 (becoming double-positive cells, DP) and, under the control of the thymic microenvironment, undergo positive selection, with positively-selected thymocytes migrating toward the medulla, where some of them will die by negative selection. Mature CD4 or CD8 single-positive thymocytes will eventually leave the thymus. **(B)** Depicts the influence of α and β-adrenergic receptors (AR) along with thymocyte differentiation in the rat thymus. Activation of α1-AR reduces the frequency of DN cells and increases DP cells. It is hypothesized that signaling through β-AR (via regulation of Thy-1 expression) modulates the selection process, reducing positive and/or increasing negative selection, and detains thymocyte differentiation, while α1-AR signaling may prevent the differentiation of positively selected cells toward the CD4^+^SP phenotype. Stellate yellow cells: cortical TECs; stellate green cells: medullary TECs; round cells with a purple nucleus: thymocytes in different stages of development; blue cells: macrophages; brown cells: dendritic cells (DCs). Reduction = – and increase = **+** Adapted from Leposavić et al. (2008) and Savino et al. (2015).

The thymus is a primary lymphoid organ responsible for the generation of T lymphocytes in vertebrates, from fish to mammals (Geenen and Savino, 2019). This process is dependent on interactions controlled by the thymic tridimensional network, composed of thymic epithelial cells (TEC), thymic dendritic cells (TDC), macrophages, fibroblasts; as well as the extracellular matrix, cytokines, chemokines, hormones; and components of the nervous system. During thymocyte differentiation, bone marrow-derived early T-cell precursors (ETP) enter the organ by the corticomedullary region of the thymic lobules and subsequently migrate to the subcapsular region. From this region, immature thymocytes, including those bearing the phenotypes TCR^–^CD3^–^CD4^–^CD8^–^ (double-negative or DN, standing for the membrane expression of CD4 and CD8) and TCR^*low*^CD3^l*ow*^CD4^+^CD8^+^ (double-positive for the same markers, or DP) migrate to the inner cortex, and then to the cortico-medullary region. Developing cells next migrate to the medullary region, where they become mature TCR^*high*^CD3^*high*^CD4^+^CD8^–^ (single-positive for CD4 or CD4SP) or TCR^*high*^CD3^*high*^CD4^–^CD8^+^ (single-positive for CD8 or CD8SP) cells (summarized in Figure 1A). Those cells ultimately migrate to the cortico-medullary region and leave the thymus to specific regions in peripheral lymphoid organs (Francelin and Verinaud, 2011; Savino et al., 2016).

The migration facilitates the encounter and the interactions of thymocytes with different components of the thymic microenvironment. For example, the TCR/CD3 complex, expressed by thymocytes, interacts with self-antigens presented by molecules of the major histocompatibility complex (MHC), expressed by microenvironmental cells, including TEC and TDC. Thymocytes bearing TCR/CD3 complexes that interact with low or medium affinity with MHC-presented self-antigens are positively selected and continue their maturation process. Conversely, thymocytes bearing TCR/CD3 complexes that interact with high affinity with MHC-presented self-antigens are negatively selected and die by apoptosis. Those selective processes prevent the exit of dysfunctional mature T cells or cells with autoimmune potential (Francelin and Verinaud, 2011; Savino et al., 2016).

As for components of the nervous system that could impact thymus physiology, nerve fibers and neurotransmitters are spread through the parenchyma, and both thymocytes and TECs express neurotransmitter receptors. This can be exemplified by the intrathymic presence of autonomic nervous system (ANS) fibers, providing noradrenergic fibers within the thymus parenchyma (Leposavić et al., 2008; Godinho-Silva et al., 2019). The main source of these nerves is the postganglionic neurons in the upper paravertebral ganglion of the sympathetic chain, predominantly from the upper cervical and stellate ganglia (Nance and Sanders, 2007; Leposavić et al., 2008). Most of the nerves are composed of noradrenergic fibers that enter the thymus through the capsule and are distributed along with the capsule and septa as fibers or following the vasculature of the connective tissue that forms the organ. Neuronal-derived norepinephrine release is modulated by presynaptic receptors of noradrenergic axonal terminals, which express the α2 adrenergic presynaptic receptors, muscarinic and nicotinic acetylcholine receptors, as well as purinergic receptors and receptors for prostaglandins (PGE2) (Haskó et al., 1995), thus illustrating a complex intrathymic control of norepinephrine release at the nerve endings.

Peptidergic innervation can also be seen within the organ, and comprise, for example, Neuropeptide Y, neurotensin, vasoactive intestinal peptide (VIP) among others (Mignini et al., 2011, 2014). Nevertheless, it is important to point out that neurotransmitters of non-neuronal origin can also be found in the thymus, and that parenchymal lymphoid and non-lymphoid cells can express the respective cognate receptors, as seen below.

## Intrathymic Expression of Non-Neuronal Neurotransmitters and Their Corresponding Receptors

As mentioned above, neurotransmitters are constitutively produced in the thymus by both autonomic innervation as well as thymic cells (Leposavić et al., 2008; Mignini et al., 2014). They comprise amino acids, monoamines, and peptides, which, as a whole, participate in the general process of T cell differentiation. Accordingly, both thymic microenvironmental cells and thymocytes express specific receptors (see Table 1).

**TABLE 1:** Intrathymic expression of non-neuronal neurotransmitters and their corresponding receptors.

Non-neuronal			Cell types expressing the		
neurotransmitter	Cell type producer		corresponding receptors		References
		
	TEC	Thymocytes	TEC	Thymocytes	
Acetylcholine	+	+	α2, α3, α5, α7, β4, ε*	α3, α5, α7, β2, β4*	Rinner et al., 1999; Kawashima and Fujii, 2004; Panneck et al., 2014
Dopamine	+	Lowlevels detected	ND	D1, D2, D3, D4, D5	Pilipović et al., 2008; Mignini et al., 2009, 2013; Lifantseva et al., 2016
Norepinephrine	+	+	α1, β1, β2**	α1, β2**	Marchetti et al., 1994; Leposavić et al., 2008; Pešić et al., 2009; Roggero et al., 2011
Serotonin	ND	+	ND	5-HT1B, 5-HT1F, 5-HT2A, 5-HT2B, 5-HT6, 5-HT7	Stefulj et al., 2000; Lifantseva et al., 2017
Vasoactive intestinal peptide	+	ND	ND	VIPR, VIP_1_R	Delgado et al., 1996a,b; Silva et al., 2006
Neuropeptide Y	+	ND	ND	Y_1_,Y_2_,Y_3_ (possibly)	Medina et al., 2000; Silva et al., 2006
Calcitonin gene related peptide	+	ND	ND	CGPR_1_	Kurz et al., 1995; Silva et al., 2006
Substance P	+	+	ND	NK-1R	Santoni et al., 2002; Silva et al., 2006
Oxytocin	+	ND	ND	OTR	Robert et al., 1991; Hansenne et al., 2005; Savino et al., 2016
Arg-Vasopressin	+	ND	ND	V1bR	Robert et al., 1991; Hansenne et al., 2005; Savino et al., 2016
Somatostatin	+	ND	sst1, sst2A	sst2A, sst3, sst4	Ferone et al., 2002; Silva et al., 2006

ND, Not determined; TEC, thymic epithelial cells; * α 2-7, β2-4, and ε, Nicotinic receptor subunits; D1-5, Doparminergic receptor; ** α1, and β1-2, Norepinephrine receptor; 5-TH1-7, Serotoninergic receptors; VIPR, Vasoactive Intestinal Peptide Receptor; Y, neuropeptide receptor; CGPR, Calcitonin Gene Related Peptide Receptor, NK-1R, Neurokinin 1-Receptor, OTR, Oxytocin Receptor, V1bR, Vasopressin V1b Receptor, sst 2-4, Somatostatin Receptor.

For example, there is an intrathymic cholinergic system represented by acetylcholine (ACh), which is synthesized and released by TECs as well as thymocytes (Rinner et al., 1999; Kawashima and Fujii, 2004; Panneck et al., 2014). These same cell types also express the corresponding cholinergic receptors, thus pointing to a non-neuronal autocrine/paracrine cholinergic circuit acting within the organ (Kawashima and Fujii, 2004). In fact, intrathymic ACh impacts thymocyte development directly by acting upon thymocyte via cholinergic receptors; or indirectly, through microenvironmental cells, with consequences on developing thymocytes (Rinner et al., 1999; Kawashima and Fujii, 2004). Actually, when thymocytes were co-cultured with TECs in the presence of ACh, a pro-apoptotic effect was observed. This effect was reversed after the treatment with a nicotinic receptor antagonist, indicating that thymic cells are responsive to cholinergic modulation (Rinner et al., 1999). Additionally, multiple nicotinic receptor subunits have been described as being expressed by thymocytes and TECs, including α2, α3, α5, α7, β4, ε in TECs and α3, α5, α7, β2, β4 in thymocytes (Mihovilovic et al., 1997; Kawashima and Fujii, 2004). The fact that both cell types express nicotinic receptor subunits reinforces the idea that ACh may be an important player in TEC-thymocyte interactions (Rinner et al., 1999; Kawashima and Fujii, 2004). The subunit expression also varies according to the cell maturation profile, as it has been shown that especially α3 and β4 are decreased in more mature single-positive thymocytes, indicating that ACh may also play a role in intrathymic T-cell differentiation (Mihovilovic et al., 1997). Moreover, an autocrine loop may take place in endothelial cells, since Ach is produced by these cells, which also express cholinergic receptors (Kawashima and Fujii, 2004). A further non-excludent hypothesis is that Ach produced by endothelial cells in the thymus act through a paracrine way, to modulate the interactions between thymocytes and endothelial cells and also TEC-thymocyte interaction.

In a second vein, the stimulation of the Ach receptor modulates nitric oxide which in turn acts by inducing vasodilation controlling permeability and blood circulation (Kawashima and Fujii, 2004).

Regarding monoamines neurotransmitters, the catecholamine system seems to be a major player in terms of the roles of neurotransmitters in thymocyte development. Norepinephrine (NE) is the main catecholamine of the ANS within the thymus. Interestingly, in addition to the neuronal input, the thymus presents a non-neuronal catecholaminergic network; thymocytes and TECs express catecholaminergic receptors and enzymes necessary for NE production, such as tyrosine hydroxylase (Leposavić et al., 2008). In this respect, inhibition of this enzyme evoked reduction in NE contents in rat thymocytes and adult thymus (Radojevíc et al., 2014).

Catecholamines secreted and released intrathymically engage with α and β adrenergic receptors expressed by thymocytes and TECs, shown in Table 1 and Figure 1B (Leposavić et al., 2008; Roggero et al., 2011). Briefly, thymocytes of adult mice and fetuses express β-AR binding sites (Singh et al., 1979). Interestingly, in the rat thymus, β2-AR, which is the most expressed adrenergic receptor in the organ, can be modulated by sexual hormones, as it was demonstrated that β2 –AR density can vary according to the marked alterations of the sex steroid hormone milieu in female rats (Marchetti et al., 1994). In a second vein, immature thymocytes in the mouse thymus express lower amounts of β-AR than their mature counterparts (Radojcic et al., 1991), suggesting that the adrenergic system participates in early T cell development through the α-AR induced signaling pathway (Kavelaars, 2002; Pešić et al., 2009). The α-AR receptors detected in the rat and human thymuses are not observed in peripheral blood mononuclear cells, suggesting that the expression of these receptors is strictly regulated during the T-cell development (Kavelaars, 2002). It is also interesting to note that non-lymphoid cells represent the major populations expressing α1-AR, even though distinct thymocyte populations could also express this receptor, mainly in immature thymocytes (Pešić et al., 2009).

Of note, phenylethanolamine N-methyltransferase (PNMT), the enzyme that converts norepinephrine to epinephrine, was also detected in the cortex and medulla of the rodent thymus, with higher density in the cortex (Warthan et al., 2002). Although the specific cell type expressing this enzyme was not detected, thymocytes would be an appropriate candidate due to tyrosine hydroxylase expression in these cells (Warthan et al., 2002; Leposavić et al., 2008). The presence of this enzyme in the thymus suggests *de novo* synthesis of epinephrine in the thymus, and that thymocytes could uptake NE and convert to epinephrine (Warthan et al., 2002; Leposavić et al., 2008). Taking into account that the affinity of epinephrine for β2-AR is much higher than NE and that β2-AR is the major β-AR subtype in the thymus (Marchetti et al., 1994), it is conceivable to think that such a conversion could enhance β2-AR driven effects upon thymocyte differentiation (Leposavić et al., 2008).

Dopamine (DA) levels were also detected in rat thymocytes, although in low levels (Pilipović et al., 2008). On the other hand, DA stores were reported in the rat thymus, located mainly in the corticomedullary and medulla of the thymic lobules, alongside vesicular monoamine transporters and thymic cells that express dopaminergic receptors (Mignini et al., 2009). This indicates that catecholamines, and especially DA, of thymic origin, can modulate the final stages of intrathymic T cell differentiation (Mignini et al., 2009, 2013; Lifantseva et al., 2016).

Immunoreactivity for the five dopamine receptor subtypes (D1, D2, D3, D4, and D5) were detected in the thymic tissue, with higher expression in cortico-medullary junction and medulla (Mignini et al., 2009, 2013). Since D1 and D3 expression is detected in the rat thymus as early as day 16 of fetal development (E16) and D5 appeared 1 day later, both DA stimulatory (D2, D3, and D4) and inhibitory (D1 and D5) pathways may be involved in the early thymus organogenesis and upon the initial events of thymocyte differentiation and migration (Lifantseva et al., 2016). Accordingly, DA receptors expression pattern is modified as thymocytes differentiate into the CD4SP or CD8SP subpopulations, and between them, suggesting that the intrathymic dopaminergic system plays a role on thymocyte lineage progression within the organ (Mignini et al., 2013).

Encompassing all monoamine neurotransmitters, thymic cells express components of the serotonergic system, as exemplified by the production of serotonin by thymocytes. Moreover, serotonergic receptors are detected in both embryonic and adult rat thymocytes (Table 1; Stefulj et al., 2000; Lifantseva et al., 2017) and their signaling seems to be related to the control of cytokine production (IL-4 and IL-2) by developing thymocytes (Shenoy et al., 2013).

The intrathymic peptidergic neurotransmitter complex, produced by thymocytes and microenvironmental cells (in addition to nerve fibers) comprises neuropeptide Y (NPY), somatostatin (SOM), substance P (SP), calcitonin-gene-related-peptide (CGRP), neurotensin, vasoactive intestinal polypeptide (VIP), PACAP (pituitary adenylate cyclase-activating peptide) as well as the oxytocin and Arg-vasopressin, which play a role in the thymus microenvironment as well as lymphocyte maturation (Silva et al., 2006; Mignini et al., 2011). In the human thymus, NPY and VIP are the major peptidergic components modulating T cell development (Medina et al., 2000; Silva et al., 2006; Mignini et al., 2011). The intracellular signaling mediated by NPY through different functional NPY receptors exerts an inhibitory chemotactic and proliferative effect on the developing thymocytes, suggesting a role for NPY in intrathymic T cell migration and emigration, which can be modified by the aging process (Medina et al., 2000). The presence of NPY^+^ fibers in the thymus associated with mast cells and macrophages, in addition to thymocytes, suggests a role for NPY in the maintenance of thymic non-lymphoid cell populations, which, in turn, contributes to thymocyte development (Mignini et al., 2011, 2014).

As regards VIP, it has been shown that it induces the differentiation of DP into CD4-SP cells, as ascertained by T cell lines (Pankhaniya et al., 1998). In a second vein, it has been demonstrated that DP and SP, but not DN thymocytes, express VIP and VIP 1 receptor (VIP1-R) genes, indicating a possible autocrine/paracrine role for VIP in thymocyte differentiation, proliferation, and survival (Delgado et al., 1996a,b). Interestingly, *in vitro* thymocyte proliferation and cytokine production were inhibited after the TCR/CD3 complex stimulation in the presence of VIP (Xin et al., 1994), although *in vivo*, effects on thymocyte proliferation were not observed following VIP treatment. However, rat thymic cells that received VIP antagonists (VIP-A1) had enhanced mitotic activity (Trejter et al., 2001), indicating that VIP effects are dependent on the thymic microenvironmental components. VIP also seems to inhibit cell death as it has also been demonstrated that both VIP, as well as PACAP27 and PACAP38 peptides, can inhibit spontaneous thymocyte death and dexamethasone-induced death through a single receptor, VIP-R (Delgado et al., 1996a). Of note is also the fact that NPY, VIP, and PACAP inhibit thymocyte chemotactic response to N-Formyl-methionyl-leucyl-phenylalanine, a well-known lymphocyte chemoattractant (Schubert and Müller, 1989).

Yet, human thymocytes express different subtypes of somatostatin receptor (SSRs) during the developing process, which are activated upon binding with their ligands, the somatostatin (SOM) produced by TECs. The intracellular pathways mediating the SOM-dependent activities are involved in the maturation and selection of T cell repertoire through regulating the proliferation and maturation process of immature thymocytes, which include their migration through thymus stroma, cytokine production, and thymic export (Ferone et al., 2002; Silva et al., 2006).

Substance P is endogenously produced in the thymus from different species by thymocytes and TECs, being mainly involved in the thymocytes rescue from spontaneous and NK-1R antagonist (SR140333)-induced apoptosis (Santoni et al., 2002; Silva et al., 2006).

Calcitonin gene-related peptide (CGRP) and its cognate receptor are also constitutively expressed in the thymus (Kurz et al., 1995). Both ligand and receptor are expressed by cells in the medulla and corticomedullary junction of the thymic lobules, suggesting the involvement of CGRP upon thymocyte proliferation during positive selection (Kurz et al., 1995; Mignini et al., 2014), although definitive functional studies are still needed.

Lastly, oxytocin and vasopressin are present in the human thymus and TECs can constitutively produce both neurohormones (Moll et al., 1988; Robert et al., 1991; Savino et al., 2016). Their function in thymocyte development is still not clear, but they seem to be involved in the control of CD8-SP cells cycle (Robert et al., 1991; Hansenne et al., 2005; Savino et al., 2016), as well as in the expression of tissue-specific antigens by the thymic epithelium, with consequent effect upon thymocyte selection process, particularly negative selection events (Geenen and Savino, 2019).

Overall, neurotransmitters exert a complex role in thymic microenvironment and, as consequence, in thymocyte differentiation. Since a diverse number of thymic cells are able to secrete and respond to those molecules, neurotransmitters can act directly on thymocytes or on stromal and endothelial cells. Hence, this data can lead us to the hypothesis that neurotransmitters affect the release of immunocompetent T-cells which will alter peripheral immune responses.

## Conclusion

Taken together, the data discussed herein unravel the complex range of interactions in the thymus that are mediated by neurotransmitters and can modulate the thymus physiology by the induction and/or inhibition of cell survival, proliferation, differentiation, and migration. Accordingly, neurotransmitters seem to provide a complex network of interactions, involving microenvironmental cells, endothelial cells and developing thymocytes ultimately modulating the intrathymic T-cell development. Conceptually, these findings lead to the notion that neurotransmitters, of both neuronal and non-neuronal origin, have distinct functions in the thymus, which impact T-cell development. In this respect, it is plausible that thymic homeostasis is partially dependent on neurotransmitters, and therefore, pathological alterations in the neurotransmitters mediated circuits in the thymus may have consequences upon normal thymocyte development. Yet, the molecular mechanisms of action and control of intrathymic production and release of these neurotransmitters remain an open space for further investigation.

## Author Contributions

All authors equally contributed to the production of this manuscript.

## Conflict of Interest

The authors declare that the research was conducted in the absence of any commercial or financial relationships that could be construed as a potential conflict of interest.
